# What can we learn from molecular dynamics simulations for GPCR drug design?

**DOI:** 10.1016/j.csbj.2014.12.002

**Published:** 2014-12-10

**Authors:** Christofer S. Tautermann, Daniel Seeliger, Jan M. Kriegl

**Affiliations:** Boehringer Ingelheim Pharma GmbH & Co. KG, Lead Identification and Optimization Support, Birkendorfer Str. 65, D-88397 Biberach a.d. Riss, Germany

**Keywords:** Molecular dynamics simulations, GPCR, Homology modeling, Water network, CC chemokine receptor 3, Muscarinic acetylcholine receptor 3

## Abstract

Recent years have seen a tremendous progress in the elucidation of experimental structural information for G-protein coupled receptors (GPCRs). Although for the vast majority of pharmaceutically relevant GPCRs structural information is still accessible only by homology models the steadily increasing amount of structural information fosters the application of structure-based drug design tools for this important class of drug targets. In this article we focus on the application of molecular dynamics (MD) simulations in GPCR drug discovery programs. Typical application scenarios of MD simulations and their scope and limitations will be described on the basis of two selected case studies, namely the binding of small molecule antagonists to the human CC chemokine receptor 3 (CCR3) and a detailed investigation of the interplay between receptor dynamics and solvation for the binding of small molecules to the human muscarinic acetylcholine receptor 3 (hM3R).

## 1 Introduction

G-protein coupled receptors (GPCRs) are key elements of eukaryotic signaling cascades. They transduce stimuli from the extracellular compartment into the interior of the cell, where further intracellular signaling events are triggered. Under physiological conditions GPCR signal transduction is initiated by their endogenous ligands, which range from lipids, fatty acids, neurotransmitters, cytokines, hormones, to metal ions or – in a figurative sense – even light. The opportunity to modify cellular signaling cascades by modulating the function of GPCRs makes them an attractive class of targets for pharmaceutical drug discovery and development efforts [1,2]. To date approximately 30% or even more of all marketed drugs target GPCRs [3,4], and still a substantial fraction of drugs that were recently approved by US regulatory authorities are GPCR drugs [5]. According to recent estimates ~ 350 GPCRs are of potential interest to treat human diseases [6]. There are still ~ 100 orphan receptors for which neither the natural ligand nor the physiological role is yet known [7].

A breakdown of the number of marketed GPCR drugs reveals that the number of unique GPCRs which are targeted is much less than what would be expected from the mere share of all marketed drugs: the GPCR drugs cover less than 10% of the entire target space which is addressed to date [5]. In other words, there are still ample opportunities to exploit hitherto unexplored GPCR targets with known ligands and function, but also orphan receptors. Besides novel molecular and cell biology techniques such as specifically engineered receptors [2] or the systematic generation of chemical probes [8–10] recent breakthroughs in the elucidation of GPCR structural information [11–13] expand the toolbox to discover and optimize novel ligands with therapeutic potential for this target class.

The increasing coverage of the GPCR phylogenetic tree with structural information offers the opportunity to apply structure-based drug design methodologies for this target class [14–17]. One approach which gained increasing attention in pharmaceutical industry over recent years is fragment-based drug discovery [18–20], which is nowadays reported to be applied also in GPCR drug discovery programs [21–25]. Despite the continuous progress in structure elucidation, the experimental determination of GPCR structures is still a cumbersome and slow process, which does not match the typical cycle times in lead optimization. Thus, only very few case studies are described in which ligand optimization is actually accompanied by experimentally solved GPCR-ligand complexes [26]. In most cases cost-efficient alternatives like homology modeling which allow rapid project support are reported [15]. Moreover, it is important to note that despite the continuous increase of solved GPCR structures for the majority of the GPCRs structural information is only accessible via homology modeling [15,27]. In many cases homology models are merely accurate enough to guide the overall direction of optimization efforts rather than to predict compound affinity [14,28,29], especially if the target to template similarity is low. Latest developments, however, show that homology models can be significantly improved by employing MD-methods [30,31], which is not surprising due to the flexible nature of GPRCs [32].

The availability of the β2 adrenergic receptor as active [33,34] and inactive [35] structure has prompted several research groups to model the activation process of GPCRs on an atomistic level by using molecular dynamics simulations [36,37]. In other studies, ligand recognition or GPCR oligomerization has been investigated by employing MD simulations (Ref [38] and references therein). All these studies have been facilitated by the availability of experimental structural information, but also by a steady increase in compute power which is provided either by continuous advancements of hardware performance including GPUs, tailored computer architectures [39], or cloud computing approaches [36]. Nowadays, several microseconds of simulation data can be collected within few days. Since the microsecond timescale marks the lower border at which important biological function such as ligand binding occurs, atomistic simulations open novel opportunities for structure-based drug discovery.

In fact, molecular dynamics (MD) simulations are being more and more used in drug design [40–42]. The notion of the importance of receptor flexibility has fostered the usage of computational tools such as MD simulations to generate ensembles of energetically accessible conformations [43,44]. Talking specifically of GPCRs, recent developments include target specific scoring functions to identify MD snapshots which still retain the typical GPCR specific conserved geometric features [30] in order to avoid unphysical decoys. Hot spots in binding pockets and, more recently, on protein–protein interfaces are being postulated by solvent MDs. For this technique small organic molecules such as propane or benzene are added to the water box, and regions of high solute density in the simulation are used as indicators of protein site druggability [45]. Recently, thermodynamic integration and/or free energy perturbation methods have gained an increasing attention in predicting relative free energies of binding [46,47].

In the following we will discuss the application of MD simulations in GPCR drug design with the help of two case studies which were performed at Boehringer Ingelheim Pharma. We selected these examples because they illustrate the different levels of information which can be utilized for drug design efforts. In the first case study binding of small molecule antagonists to the human CC chemokine receptor 3 (CCR3) was investigated. Since to date no crystal structure of CCR3 is reported we generated homology models to provide structural information for a medicinal chemistry program. Complexes with manually modeled ligand binding poses were subjected to MD simulations to check the integrity of the binding pose. The final model was utilized to rationalize rodent selectivity data. The second case study addresses the binding of small molecule ligands to the human muscarinic acetylcholine receptor 3 (hM3R). In this case, detailed experimental structural information has been available which enabled us to investigate the interplay between receptor dynamics, water networks, and ligand–receptor interactions by MD simulations on a very detailed level.

## 2 Computational methods

### 2.1 Homology modeling and ligand placement for CCR3

CCR3 homology models were generated based on several templates. In the following we will describe the homology modeling procedure employing the X-ray structure of human CC chemokine receptor 5 (CCR5) complexed with maraviroc as a template (PDB code 4MBS). This structure was solved in 2013 [48]. In order to enhance crystallization, a rubredoxin entity was fused to intracellular loop 3. To generate a model of the human CCR3 receptor, a sequence alignment between the template X-ray structure and the target hCCR3 sequence was done in MOE [49] employing the BLOSUM50 matrix. By this the exact location of the rubredoxin insertion could be identified, and rubredoxin was removed manually from the template structure. The employed sequence alignment is shown in the Supplementary material (Fig. S1), revealing a target/template sequence identity of more than 50%. The comparative modeling step was done with Modeller [50,51], a standard homology modeling tool. Finally a stepwise optimization of the structure was performed. In a first step the two loose ends caused by the cut of rubredoxin were manually connected and the whole receptor was protonated at pH 7.4 by the protonate3D procedure as implemented in MOE. In a next step an energy optimization of the receptor with high tethers on the heavy atoms was carried out (tether 1000). The tethers were then reduced to 100 and 10. In the final optimization step the side chains were energy minimized with fixed protein backbone atoms. All minimizations were done with the MMFF94x force field as implemented in MOE. The ligand (structure shown in Fig. 2A) was placed manually into the receptor. First, an ensemble of low energy conformations was generated. These conformations were then manually docked into the receptor such that the ionic interaction between the positively charged center of the ligand and the E287^7.39^ on transmembrane helix (TM) 7 was enforced. Conformations which caused major clashes were discarded, and checks like the shape overlay with maraviroc in hCCR5 or the formation of an additional ionic interaction with H97^2.67^ (for details we refer to ref. [52]) were employed to identify the most plausible pose. This pose underwent stepwise geometry optimization (as described before) and was ultimately subjected to a full equilibration and a 60 ns production run (MD setup as described below) to yield the binding mode shown in Fig. 3.

### 2.2 Molecular dynamics simulations

Molecular dynamics simulations were carried out using the gromacs-4.5 package [53]. The simulation system consists of the aforementioned CCR3 homology model or the hM3R model [54] with bound tiotropium (derived from PDB 4DAJ) embedded in DMPC lipid bilayer [55] and solvated in water. NaCl was added to achieve a 150 mM salt concentration. The amber99sb-ildn* force field [56,57], the SPC/E water model [58] and ion parameters from Joung et al. were used [59]. Force field parameters for the small molecule ligands were obtained according to the generalized amber force field (GAFF) procedure [60] with partial charges derived from quantum chemical calculations with Gaussian09 [61] at a HF/6-31G* level of theory. Amber topologies for the ligands were converted to gromacs format using acpype [62]. The membrane simulation system was built with g_membed [63]. After energy minimization, 50 ps equilibration with positional restraints on heavy atoms, and 1 ns equilibration with position restraints in z-direction on the phosphor atoms of DMPC followed. Trajectories were subsequently collected at 310 K with standard NPT ensemble settings (thermostat: velocity-rescaling [64,65]; barostat: Parrinello-Rahman [66], semi-isotropic coupling). Electrostatic interactions were calculated at every step with the particle-mesh Ewald method [67], short-range repulsive and attractive dispersion interactions were simultaneously described by a Lennard-Jones potential, which was cut off at 1.0 nm. The SETTLE [68] algorithm was used to constrain bonds and angles of water molecules; LINCS [69] was used for all other bonds. Virtual sites [70] were introduced to remove other fast vibrating degrees of freedom, allowing a time step of 4 fs. In the hM3R case five trajectories (400 ns each) were recorded for every system. Individual analyses were always done based on the averages of the 5 independent trajectories per system.

## 3 Case Study 1: binding of small molecules to the CC chemokine receptor-3

CCR3 is a member of the chemokine receptor family. One of its endogenous ligands, the chemokine eotaxin-1/CCL11, is a chemoattractive protein which has been identified to recruit eosinophils under allergic conditions and binds exclusively to CCR3. It is therefore postulated that antagonizing CCR3 with small molecules is a viable approach to treat allergic diseases such as asthma, for which numerous in vivo studies suggest that they are closely linked to eosinophilia [71,72].

**Fig. 1:**
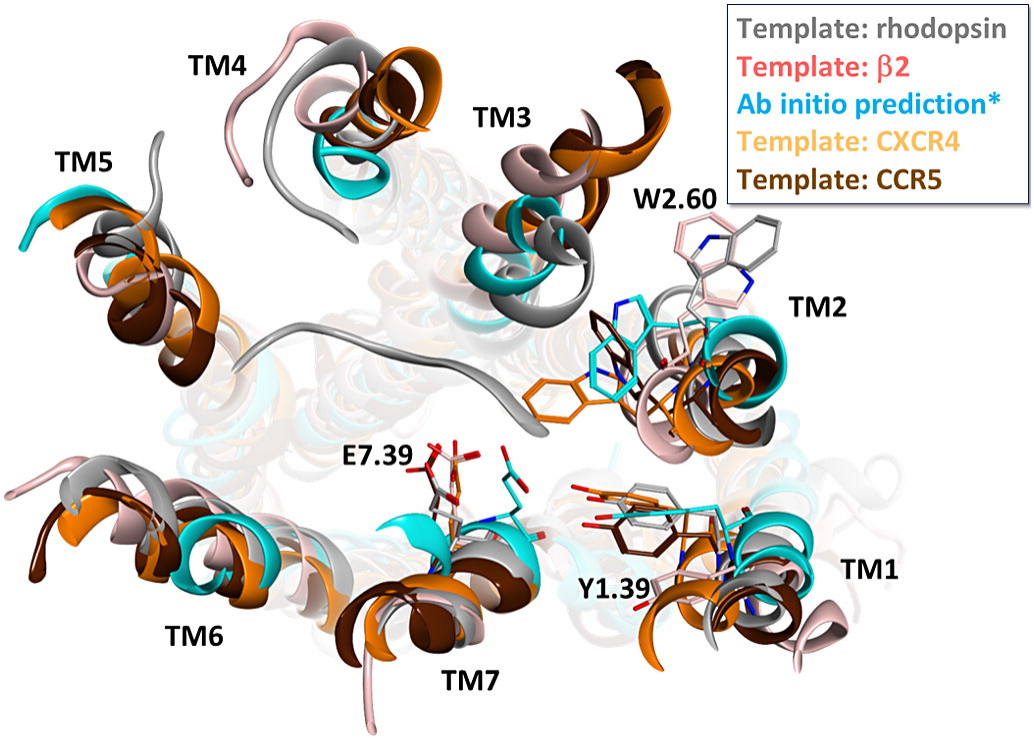
Multiple versions of the CCR3 receptor homology model, color-coded according to the template that was utilized. Top view from the extracellular side.

When optimizing antagonists for their potency on CCR3 in binding and functional cellular assays we observed a pronounced selectivity versus rodent receptor orthologues [52]. To explain the selectivity profiles and eventually identify opportunities to optimize potency especially on mouse and/or rat CCR3 we constructed a homology model of CCR3. Over the time the homology model underwent several refinements. They were triggered either by the release of a novel GPCR structure with a higher sequence similarity to CCR3 or – in one instance – by the availability of an ab-initio algorithm to predict GPCR 3D structures [73]. Fig. 1 shows an overlay of the different models, color-coded according to the template or modeling technique that was employed. In all models, E287^7.39^ (the superscript corresponds to the Weinstein–Ballesteros numbering scheme) on TM7, which is postulated as one key anchoring residue for small molecule CCR3 ligands [74] and which is highly conserved among other chemokine receptors and across species [75], points towards the interior of the transmembrane cavity in a very similar manner. The same observation was made e.g. for Y41^1.39^ on TM1. The largest differences between the individual models were observed for TM2, which underwent in total a 90° rotation throughout all model versions. When rhodopsin or β2 was used as template structure, W90^2.60^ protrudes into the lipid bilayer. We were not able to generate a plausible ligand binding mode with these two models since the cavity between TM2 and TM3 is not well defined. In the models that were either generated ab-initio or from closer related templates (CXCR4 and CCR5), W90^2.60^ lines up the TM cavity such that a hydrophobic patch is offered for ligands to interact with. A closer inspection of the CXCR4 and CCR5 X-ray structures shows that TM2 displays a helical bulge in the extracellular part, thus causing this 90° rotation of the upper part, which is different to β2/rhodopsin based models.

### 3.1 Results of Case Study 1

**Fig. 2:**
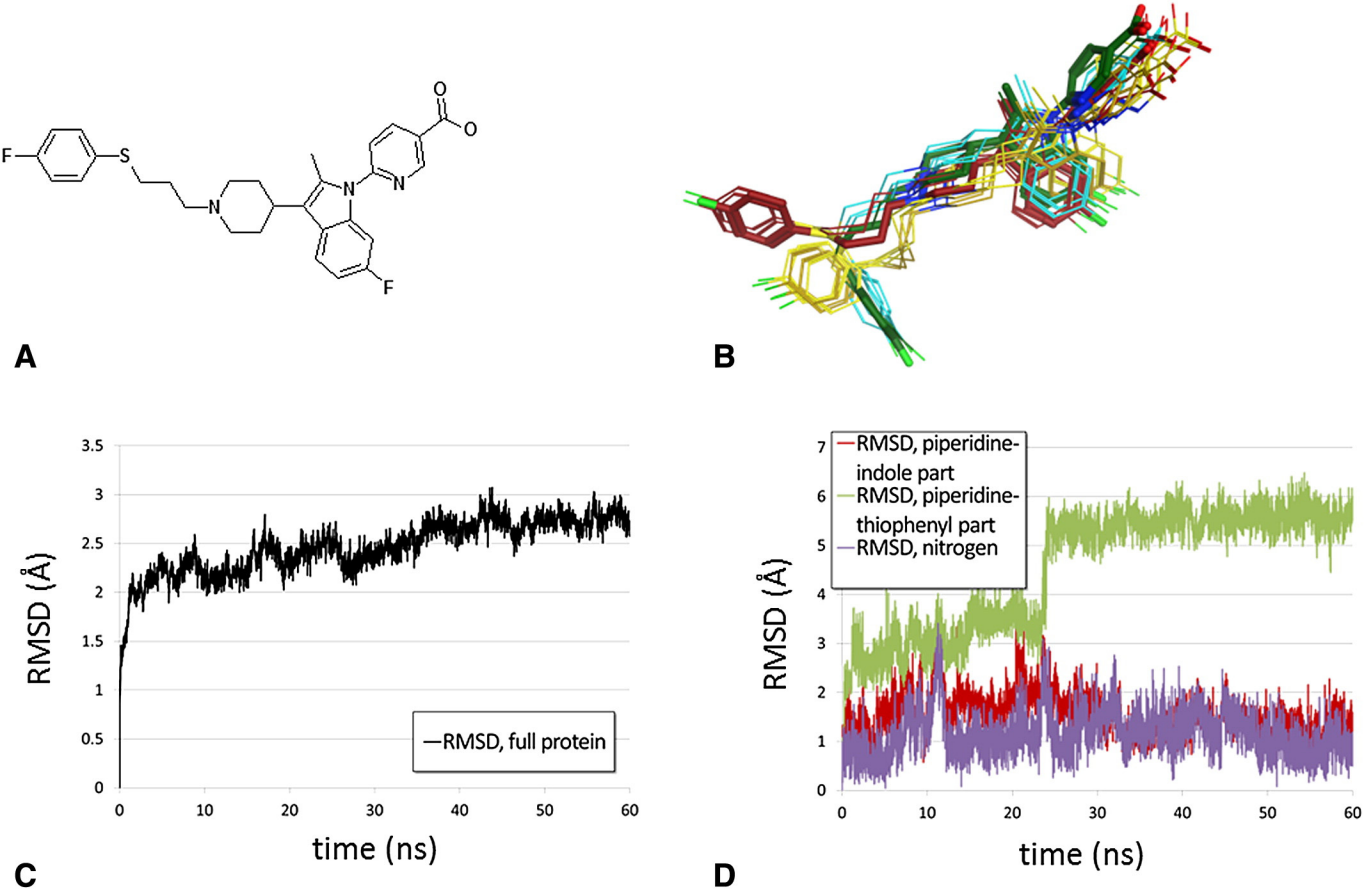
A) 2D representation of the molecule that was embedded in CCR3. B) Conformations that were visited during the simulation. The starting conformation is shown in dark red, the final conformation in dark green. C) RMSD plot for the full protein. D) RMSD plots for certain parts of the ligand, illustrating the flexibility of these parts throughout the simulation. In panels C) and D) all RMSD values are calculated with respect to the starting geometry.

**Fig. 3:**
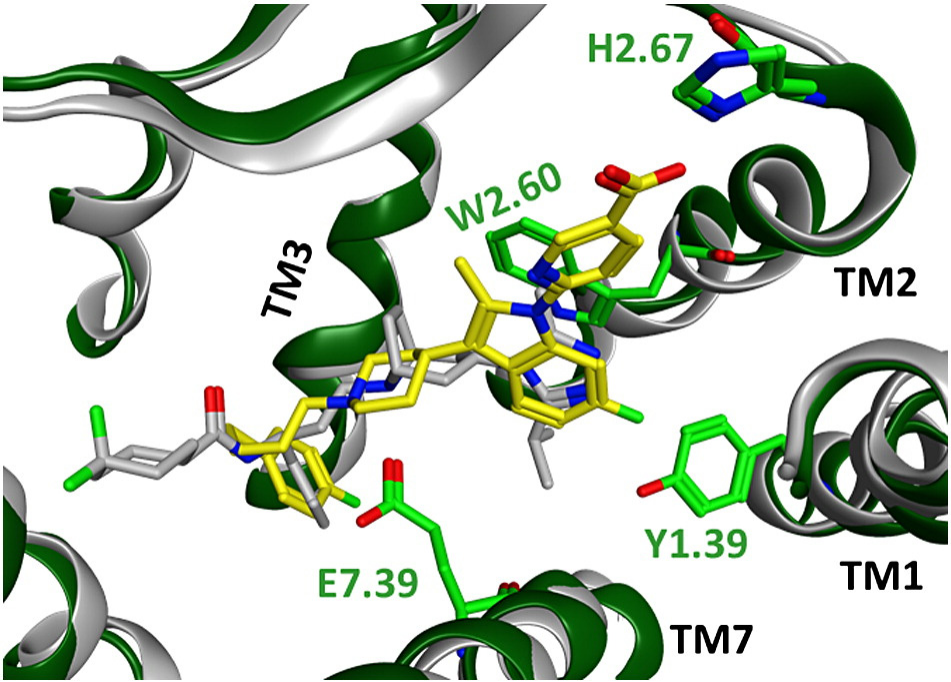
Final modeled binding mode for molecule shown in Fig. 2A. CCR3 and key residues are shown in green. For comparison, CCR5 in complex with maraviroc (PDB entry 4MBS) is shown too (gray).

We manually docked a compound (Fig. 2A) carrying the essential molecular topology and key functional groups of one of our CCR3 antagonists (ref [76]) into the CCR5-derived homology model as described above. In the initial pose ionic interactions with E287^7.39^ and H97^2.67^ and a hydrophobic interaction with W90^2.60^ were established. In addition, the hydrophobic phenylsulfanylpropyl tail pointed to a region between helices 4 and 5 (Fig. 2B, conformations colored in red). The complex was subsequently submitted to a 60 ns MD simulation to check the stability of the proposed binding mode. The entire complex remained stable during the simulation, as indicated by the RMSD plot (Fig. 2C). The ligand did not show major rearrangements such as complete dissociation from the transmembrane cavity or flips around the center of the molecule. In particular, the positioning of the piperidine-indole part, which is engaged in directed interactions with receptor residues, did not vary substantially (c.f. Fig. 2D). On the other hand, the more flexible phenylsulfanylpropyl moiety fluctuated between various orientations. This is reflected in the corresponding RMSD plots (Fig. 2C). After approximately 25 ns the tail adopted a conformation in which it protruded deeply into the interior of the TM cavity, in close analogy to the X-ray binding mode of maraviroc in CCR5 (PDB entry 4MBS [48]). This conformation remained stable over the remaining 35 ns of the simulations. Representative snapshots are shown in cyan and green (Fig. 2B). After minimization of the last snapshot of the trajectory we ultimately came up with the pose shown in Fig. 3. This pose was utilized as template to place close analogs into the receptor and ultimately to rationalize species selectivity that we observed in this particular structural class. For more details we refer to ref. [52].

### 3.2 Discussion of Case Study 1

A retrospective analysis of the modeling procedure and the outcome of the MD simulations highlight two important aspects: first, the choice of the template for homology modeling efforts has a crucial impact on the quality of the results and hypotheses which are derived therefrom. Although the GPCR structures solved to date show a high degree of analogy in the TM region [13], subtle structural features such as unusual helix turns or side chain orientations can be expected to be accurately modeled only when the template for homology modeling is sufficiently close. Even with a model that was derived from the sequence-wise close CCR5 template we observed that the modeled binding mode underwent some changes during an MD simulation. Without further experimental information it is challenging (if not impossible) to pick the relevant binding mode from an MD trajectory alone. In that respect the MD simulation helped us to build up confidence in the general orientation of the ligand inside the TM cavity and the directed (ionic) interactions that are formed. A true validation of the binding mode can only be established by experimental data. In our case, we found agreement with species selectivity data. A more rigorous validation would be provided by site-directed mutagenesis data.

## 4 Case Study 2: binding of small molecules to hM3R

**Fig. 4:**
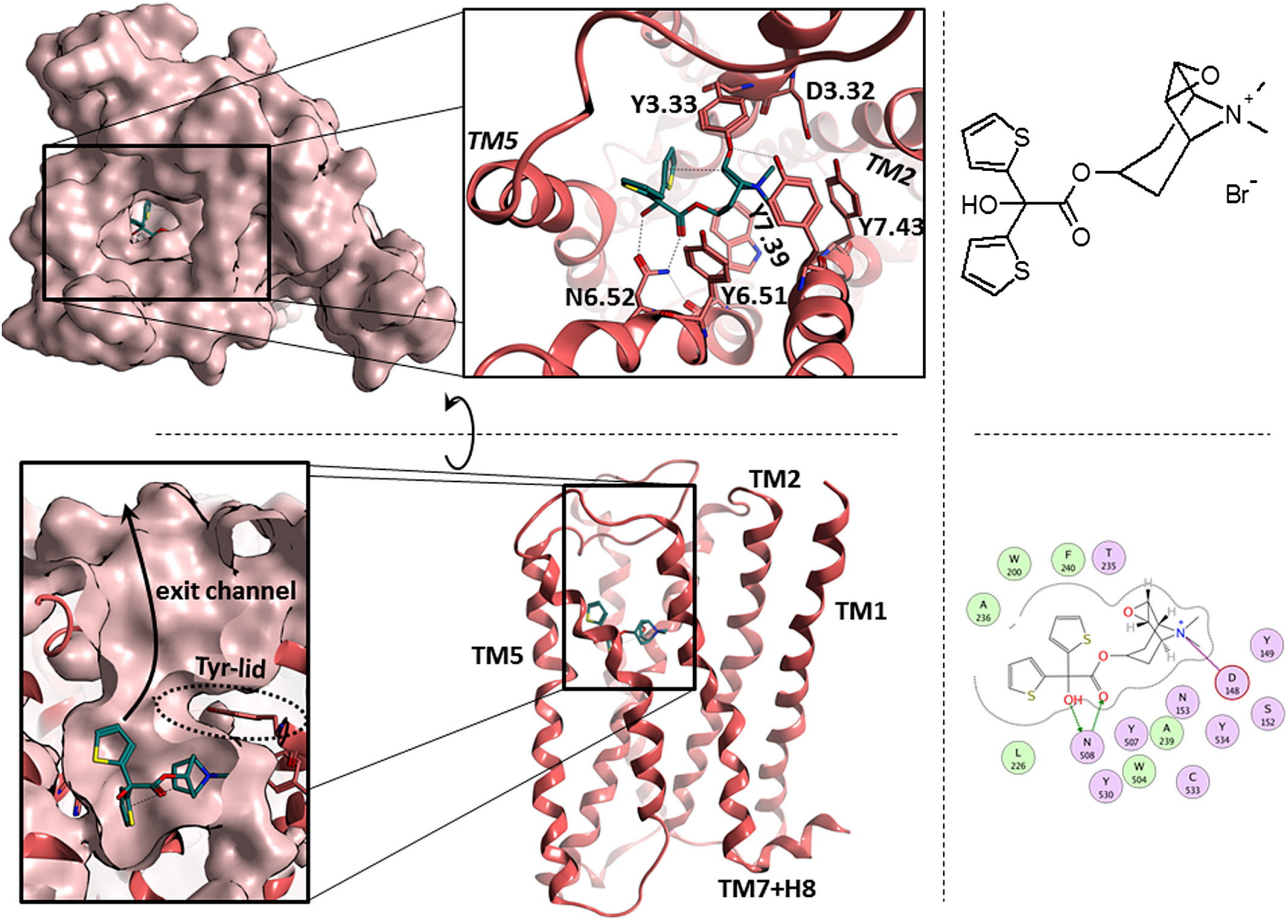
Binding mode of tiotropium (cyan) in hM3R. Top left: top view from the extracellular side. Bottom left: side view with TM6 and TM7 in front. Right: structure and 2D-interaction plot of tiotropium.

Bronchodilators are central to symptom management in airway diseases like asthma and COPD. While short-acting medications are used for immediate symptom relief, long-acting drugs are essential for disease control and maintenance therapy [77,78]. Currently two major classes of long-acting bronchodilators are available: muscarinic antagonists and β2-adrenergic agonists. The use of long-acting muscarinic antagonists (LAMAs), like tiotropium, is the mainstay of current COPD treatment [77,78]. The structure of the complex of tiotropium and rat muscarinic M3 receptor (M3R) has been solved in 2012 [79], and this makes hM3R a well suited target for structure based drug design investigations. The binding mode of tiotropium in a homology model of hM3R is shown in Fig. 4. (The homology model of the human receptor is very closely based on the X-ray structure of rat M3R and has already been described elsewhere [54].) Tiotropium is binding in a deeply buried site in the receptor, which is accessed through a narrow channel. In the binding site the positively charged epoxytropane head group of the ligand is coordinated into an aromatic/ionic cage consisting of D148^3.32^, Y149^3.33^, W504^6.48^, Y507^6.51^, Y530^7.39^, and Y534^7.43^ by ionic and aromatic interactions. Remarkably Y149^3.33^, Y507^6.51^, and Y530^7.39^ form a “lid” above tiotropium which blocks its way through the exit channel. In addition the ligand forms a strong double hydrogen bond to N508^6.52^, and the thiophene groups fit into a hydrophobic crevice formed by W200^4^^.57^, L226^5.33/EL2^, T232^5.39^, A236^5.43^, and A239^5.46^. Altogether, the ligand is very tightly bound, making all kinds of interactions – hydrophilic, hydrophobic and ionic – thus leading to a very high potency of about 10 pM [54].

In a recent study we have published residence times and binding affinities of tiotropium and related compounds to hM3R and mutated variants thereof [54]. The goal of the investigation was the elucidation of the key structural elements of tiotropium which leads to the very slow off-rate from hM3R. In the course of the study about 30 single point mutants have been generated, and the dissociation rates of tiotropium from the mutated receptors have been recorded. In most cases K_i_ values and the corresponding off-rates are directly related to each other (i.e., k_off_ = const ∗ K_i_), but mutation of residues along the exit channel accelerates the dissociation tremendously. Single amino acid mutation of Y149^3.33^, Y507^6.51^, N508^6.52^, or Y530^7.39^ results in off-rates which deviate from linearity by more than one order of magnitude. Modification of the ligand only leads to unexpected behavior if the hydroxy group is modified. As shown in Fig. 4 the hydroxy-group directly interacts with N508^6.52^, thus prompting the assumption that the double hydrogen bond is a decisive structural element for long acting antimuscarinic drugs. To rationalize the effect of the N508^6.52^A mutation several MD simulations have been performed. From them it has been proposed that the interaction of the ligand with N508^6.52^ works like a snap-lock. Once the interaction is formed it keeps tiotropium in place and prevents its translation towards the exit-channel. These investigations have been a first step to understand the experimental findings on a molecular basis. Additional, more extensive simulations for closely related ligands are the subject of the present study. The systems that were investigated are described in Table 1. For each system a total of 2 μs simulation data was collected from five independent trajectories of 400 ns length.

### 4.1 Results of Case Study 2

MD trajectories were analyzed by focusing on various different receptor regions. Therefore the description of our observations will be split into four sections: (i) changes of the tyrosine cage, (ii) changes of the ligand–receptor interactions, (iii) exit channel flexibility, and (iv) water densities and networks. In the discussion the observations will be put into context to obtain a clear picture of the changes of receptor dynamics upon mutation of receptor or ligand.

#### 4.1.1 Changes in the tyrosine cage

**Fig. 5:**
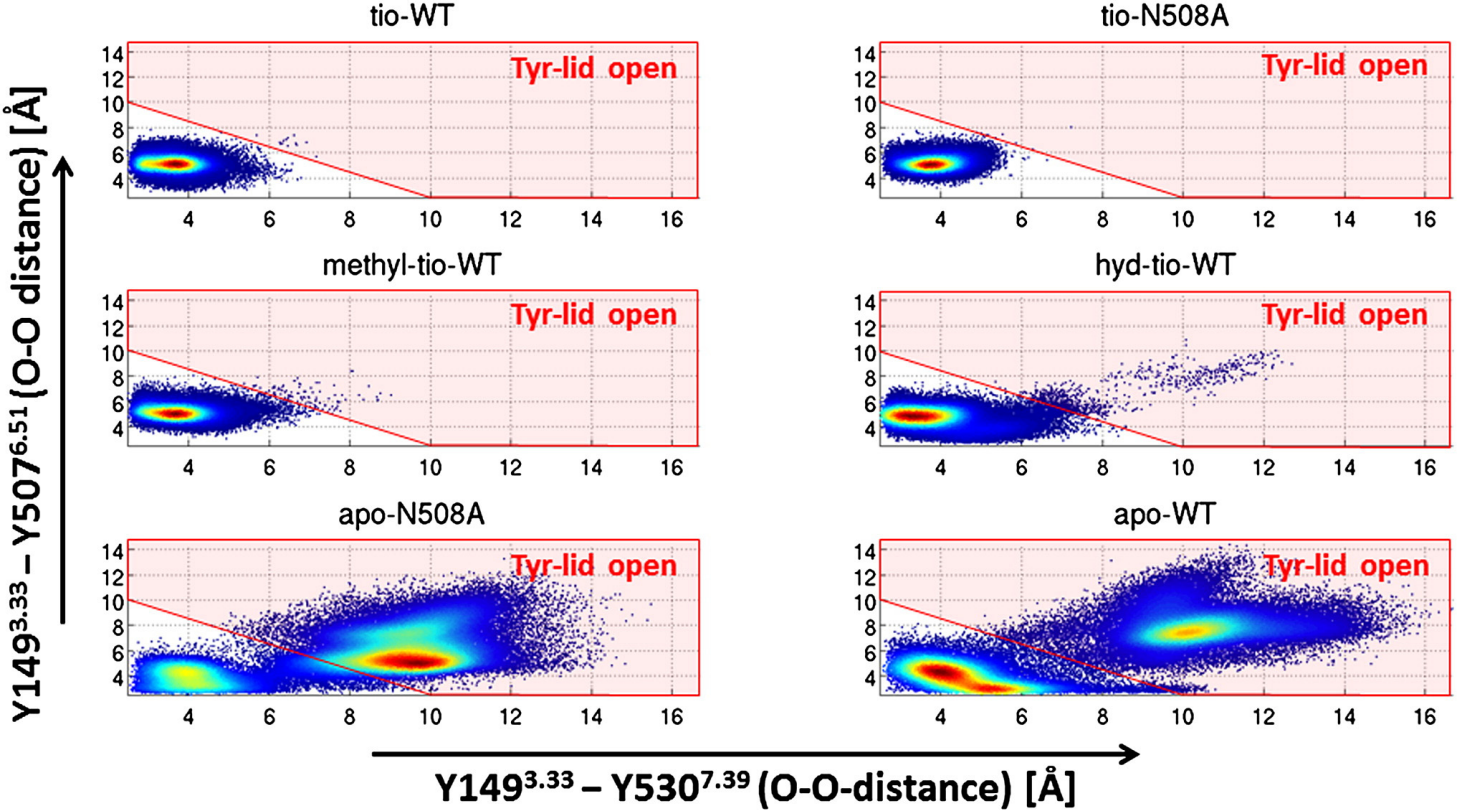
Correlation plots for 100.000 snapshots of all trajectories. Red shaded areas correspond to snapshot conformations, where the tyrosine lid opened by breaking at least one of the tyrosine hydrogen–bond interactions.

As described in the Introduction section and displayed in Fig. 4, the positively charged head group is surrounded by several tyrosine residues. The three most important tyrosines in dissociation experiments (Y149^3.33^, Y507^6.51^, and Y530^7.39^) form a lid above the ligand. This lid has to break up for ligand dissociation. In the case of tiotropium binding to wild type hM3R this lid is tightly closed [54]. In the apo-WT simulation it has been shown that the lid is open in a significant number of snapshots and thus enables quick ligand association. The correlations of movements of the lid-tyrosines in the systems under consideration are shown in Fig. 5. Ligand binding stabilizes the lid in a closed position for all ligands investigated, and in both apo simulations open as well as closed conformations are found. Surprisingly the apo-WT simulation still has the larger fraction of closed lid structures, whereas the apo simulation of the mutated receptor (apo-N508^6.52^A) shows a predominant population of snapshots with an open lid.

**Fig. 6:**
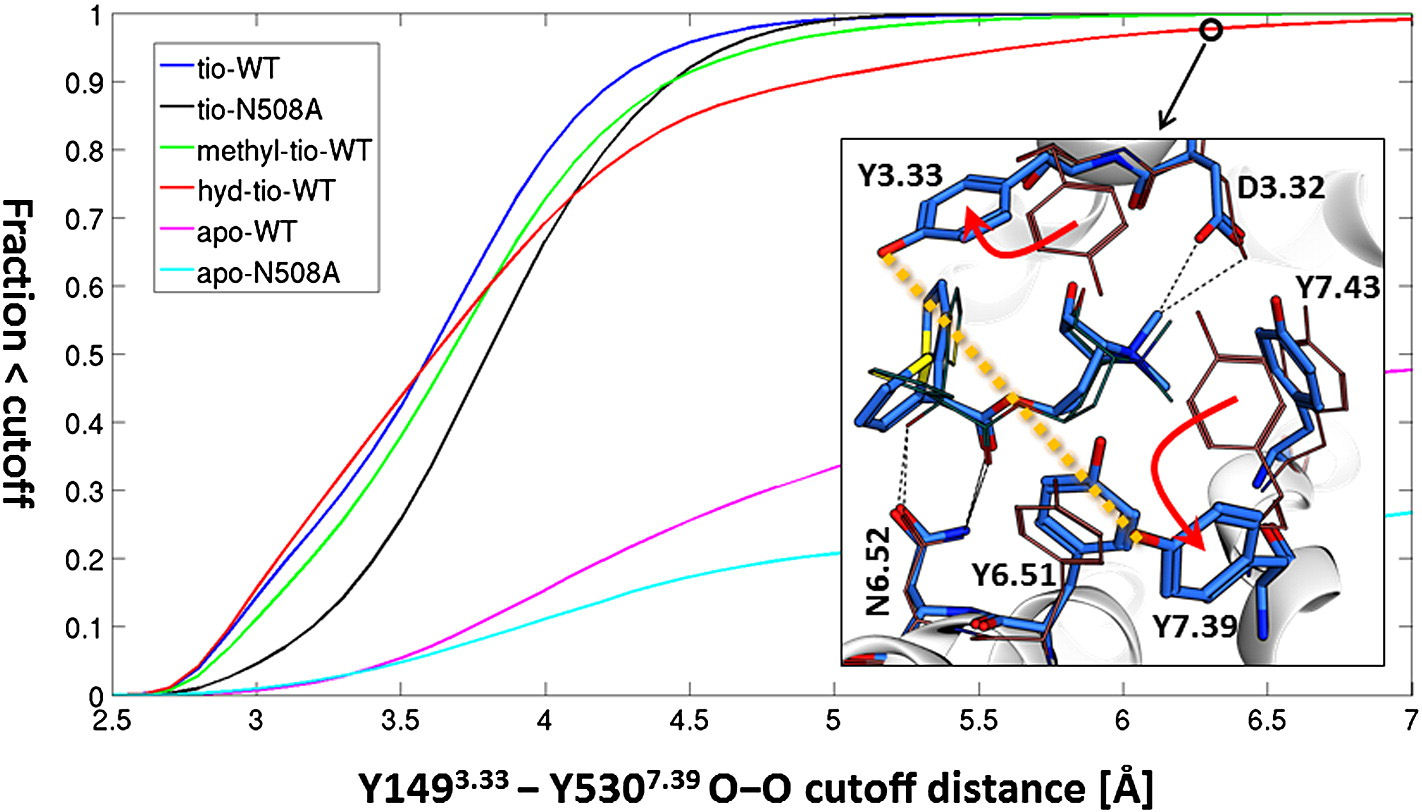
Cumulative distribution function of the Y149^3.33^–Y530^7.39^ (oxygen–oxygen) distance (orange dashed line in the insert). Inset: snapshot from the hyd-WT simulation (blue sticks) overlaid with the M3R crystal structure (orange lines). The red arrows mark lid-opening movements of Y149^3.33^ and Y530^7.39^.

In tiotropium bound simulations the tyrosine lid is very stable showing hardly any opening events. However, the distance between Y149^3.33^ and Y530^7.39^ is slightly elongated in the tio-N508^6.52^A simulation compared to tio-WT, as displayed in Fig. 6. Upon binding of methyl-tio or hyd-tio the tyrosines are much more flexible. Especially for hyd-tio the tyrosine lid is open in many snapshots. An example for a snapshot with an open lid configuration is shown in the inset in Fig. 6. We find both tyrosines (Y149^3.33^ and Y530^7.39^) in a different rotameric state compared to the M3R X-ray structure, and the ligand is directly accessible by the solvent.

To summarize the observations for the tyrosine lid flexibility: in apo simulations a dynamic equilibrium between open and closed lids is observed. Ligand binding stabilizes the tyrosine lid, however, ligands without the OH-group are not able to stabilize the lid over the full simulation time to the same extent as tiotropium.

#### 4.1.2 Changes in the ligand–receptor interactions

**Fig. 7:**
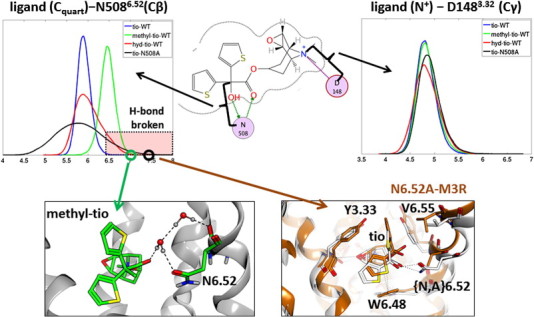
Top: Distances of the ligands to the interaction partners on the receptor side. Bottom: snapshot from the methyl-WT simulation, where the ligand–N508^6.52^ hydrogen bond is broken (left) and snapshot from the tio-N508^6.52^A simulation to demonstrate the shift of tiotropium towards the exit channel (white structure: X-ray, brown: snapshot from the tio-N508^6.52^A simulation).

Tiotropium is strongly bound to hM3R by ionic interactions with the charged epoxytropane head group, by hydrogen bonds with the ester carbonyl group as well as the hydroxy group, and by lipophilic interactions with the two thiophenes. In this chapter we investigate the changes of tiotropium movement if the hydrogen bonds are weakened or even entirely removed. In Fig. 7 the distances of the charged nitrogen of the ligand to D148^3.32^ are monitored as a measure for the flexibility of the head group. It can be observed that neither removal of the hydroxy group of tiotropium nor the N508^6.52^A mutation of the receptor has any significant effect on the distance of the ionic interaction. Not surprisingly, the mobility of the quaternary carbon of the ligand is strongly affected by distorting the double hydrogen bond. We plot the distance to the Cβ atom of N508^6.52^ (we chose to use the Cβ to be able also to include tio-N508^6.52^A in the analysis) as a measure for the flexibility of the bis-thiophene substructure in relation to the decisive N508^6.52^ interaction partner. Although both modified ligands, i.e., hyd-tio and methyl-tio, still do have the ability to form hydrogen bonds to N508^6.52^, these bonds are often strongly elongated or even broken. Especially methyl-tio shows very long distances, where the hydrogen bond is mediated by an additional water molecule, as shown in Fig. 7. The movements of tiotropium in N508^6.52^A-hM3R reveal a high flexibility due to the lack of the hydrogen bond partner N508^6.52^. Tiotropium's hydroxy group forms an intramolecular hydrogen bond to the ester carbonyl group (Fig. S2, Supplementary material). Calculation of the RMSF of different parts of the molecule confirms this observation, as shown in Fig. S5 in the Supplementary material. Additional analyses show that the quaternary carbon is located much more towards the extracellular part of the exit channel compared to the tio-WT simulation (Fig. S6, Supplementary material). A snapshot to illustrate this ready-to-exit behavior is also shown in Fig. 7.

To summarize the observations for the ligand–receptor interactions: for the modified ligands (hyd-tio and methyl-tio) the hydrogen bond between the ligand and receptor is elongated and especially for methyl-tio often broken. Tiotropium in N508^6.52^A-hM3R reveals pronounced dislocations of the bis-thiophene substructure of the molecule in the receptor and is pushing towards the exit channel.

#### 4.1.3 Flexibility of the entry/exit channel

**Fig. 8:**
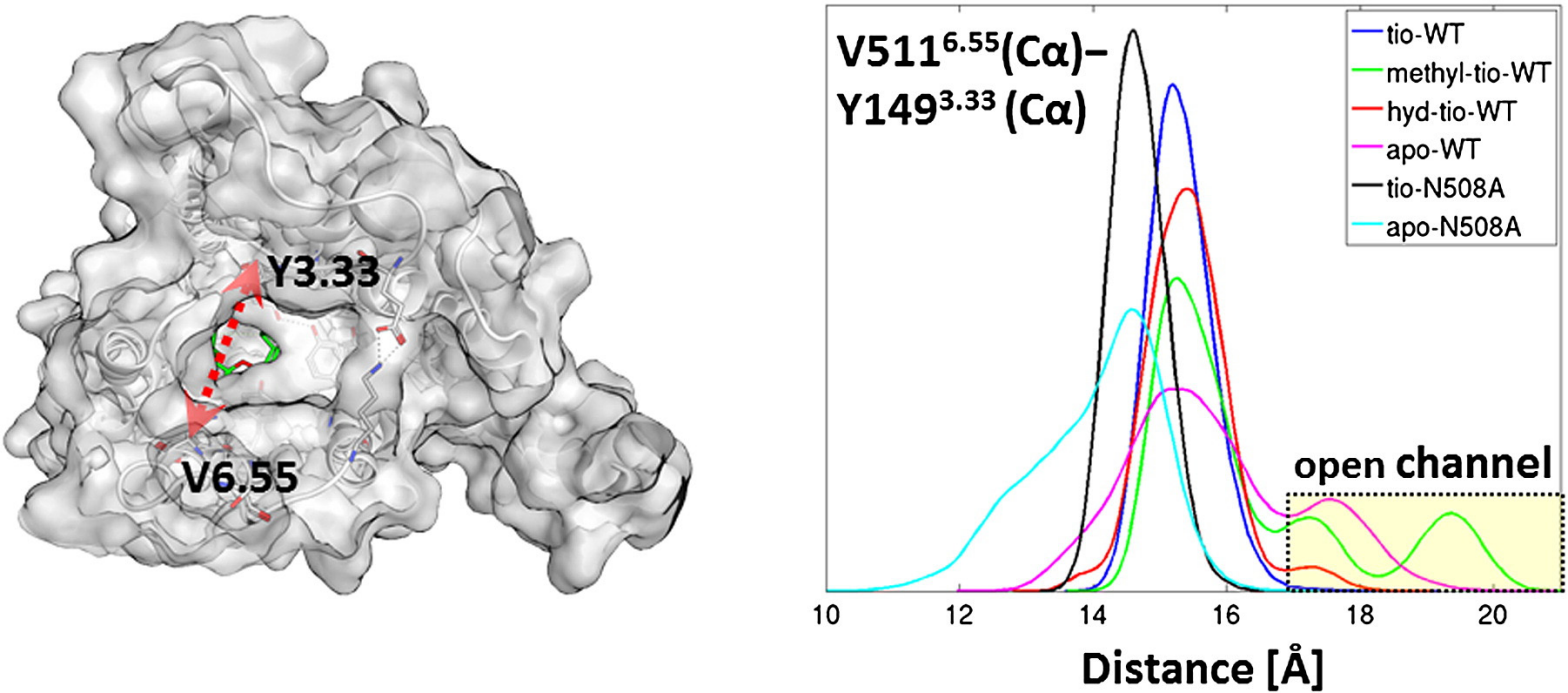
Left: location of the Cα atoms of V511^6.55^ and Y149^3.33^ as indicator for the diameter of the exit channel. Right: distribution functions of the distances between the Cα atoms of V511^6.55^ and Y149^3.33^.

So far only the direct binding site of tiotropium has been investigated. Considerations of dissociation dynamics also need to include the investigation of the dissociation pathway. As shown in Fig. 4, the binding site is accessed through a narrow tunnel. We investigate the accessibility of the entry channel by monitoring the distance of the Cα atoms of V511^6.55^ and Y149^3.33^. These two amino acids are located at opposing locations of the channel. Their location is shown in Fig. 8.

**Fig. 9:**
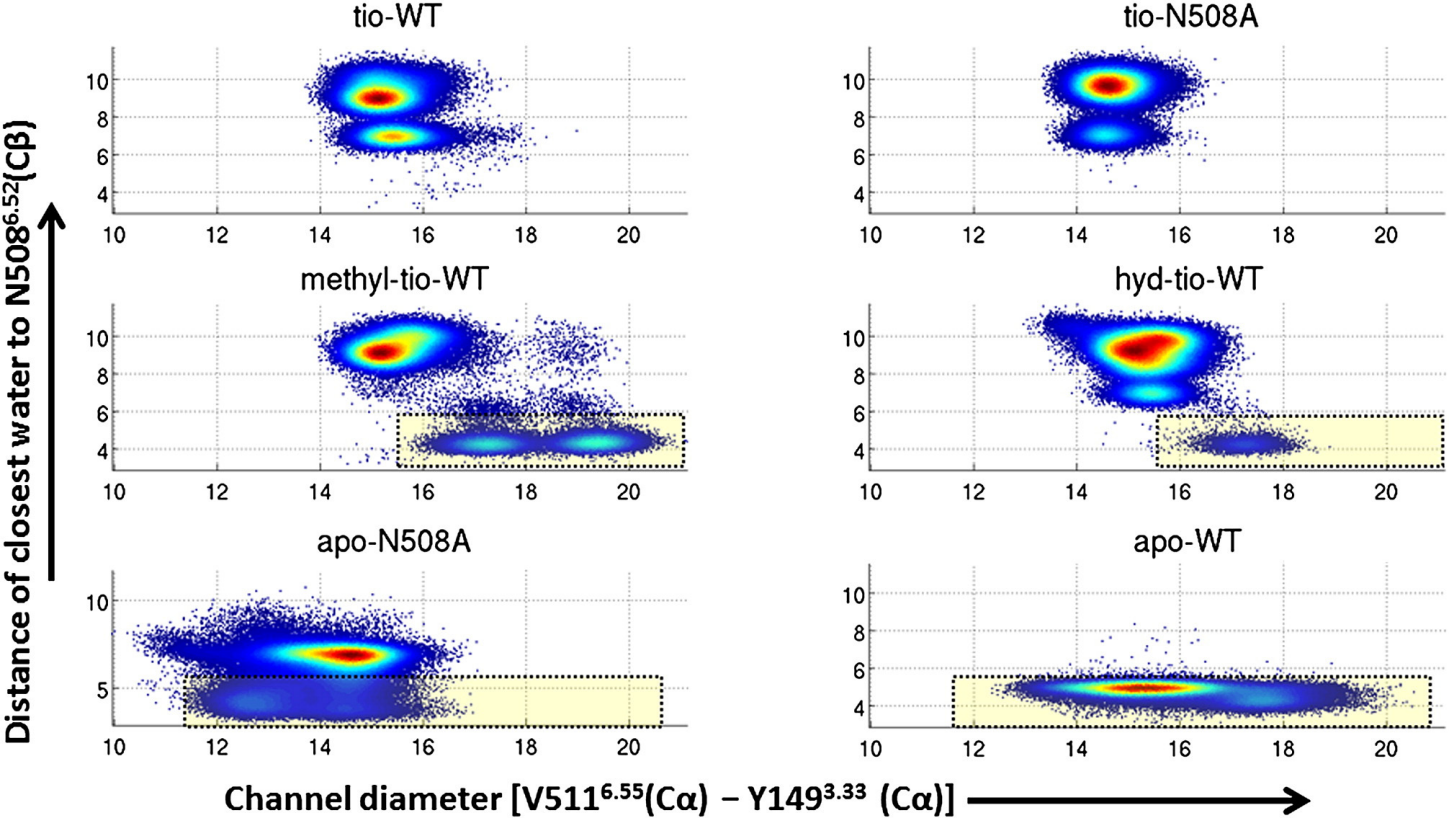
Density plots of the correlation between the channel diameter (V511^6.55^–Y149^3.33^) and the distance of the closest water to the Cβ of N508^6.52^. The yellow boxes mark the regions of direct interaction of N508^6.52^ with water.

The distribution functions of the distances show some very surprising results. Tiotropium does not allow too much flexibility of the exit channel. When simulating methyl-tio or hyd-tio in WT hM3R, the picture changes entirely, because these ligands induce open-channel states. Especially in the methyl-tio-WT simulation very large values for the diameter of the channel are observed. Similar open-channel structures are observed in the apo-WT simulation. In sharp contrast to that, the apo-N508^6.52^A simulation shows a preference for a tightly closed channel. The difference between the open state in methyl-tio-WT and apo-N508^6.52^A can be as large as 10 Å. In line with this, the channel is also slightly tighter closed in the tio-N508^6.52^A simulation than in the tio-WT simulation. Fig. 9 shows the correlation of the channel diameter and the solvation of N508^6.52^, which sits at the bottom of the channel. In WT hM3R tiotropium prevents N508^6.52^ from being solvated. A closer water site can be observed at a distance of about 7 Å. Removal of tiotropium's hydroxy group allows water to access the exit channel and to directly interact with N508^6.52^. Therefore, the close interaction is only feasible if the channel opens up — as shown in Fig. 9 (middle panel). For methyl-tio there are two distinct open states of the channel, where both allow a solvation of N508^6.52^ and with that also water insertion into the ligand-N508^6.52^ hydrogen bond. When observing the trajectories over time (Fig. S3, Supplementary material), the opening and closing motions resemble a “breathing” like behavior of the receptor. Also for hyd-tio the solvation of N508^6.52^ comes with an opening of the channel, but no distinct second (widely) open state is observed. In the WT-apo simulation N508^6.52^ is, as expected, always solvated, independently from the opening state of the channel. The simulations of the N508^6.52^A mutants have to be interpreted with caution, because the hydrophilic N508^6.52^ is substituted by an apolar amino acid, which does not require solvation to achieve a low free energy. Tiotropium bound N508^6.52^A-hM3R shows a similar pattern to tiotropium bound WT, but apo-N508^6.52^A completely deviates from apo-WT. First, the channel is always closed and second, A508^6.52^ is solvated only in a small fraction of the snapshots. All these observations strongly suggest that the opening and closing of the channel are directly connected to the water network at the extracellular surface of the receptor.

To summarize the observations for the channel flexibility: the N508^6.52^A variants of the receptor induce a more tightly closed conformation of the exit channel. This is caused by the lack of the water network required for the solvation of N508^6.52^. Tiotropium stabilizes the channel in a closed state, thus preventing water to interfere with the double hydrogen bond between N508^6.52^ and the ligand. Methyl-tio and hyd-tio enable openings of the channel, always accompanied by the entry of water, which competes with the ligand–N508^6.52^ hydrogen bond.

#### 4.1.4 Water densities and networks

**Fig. 10:**
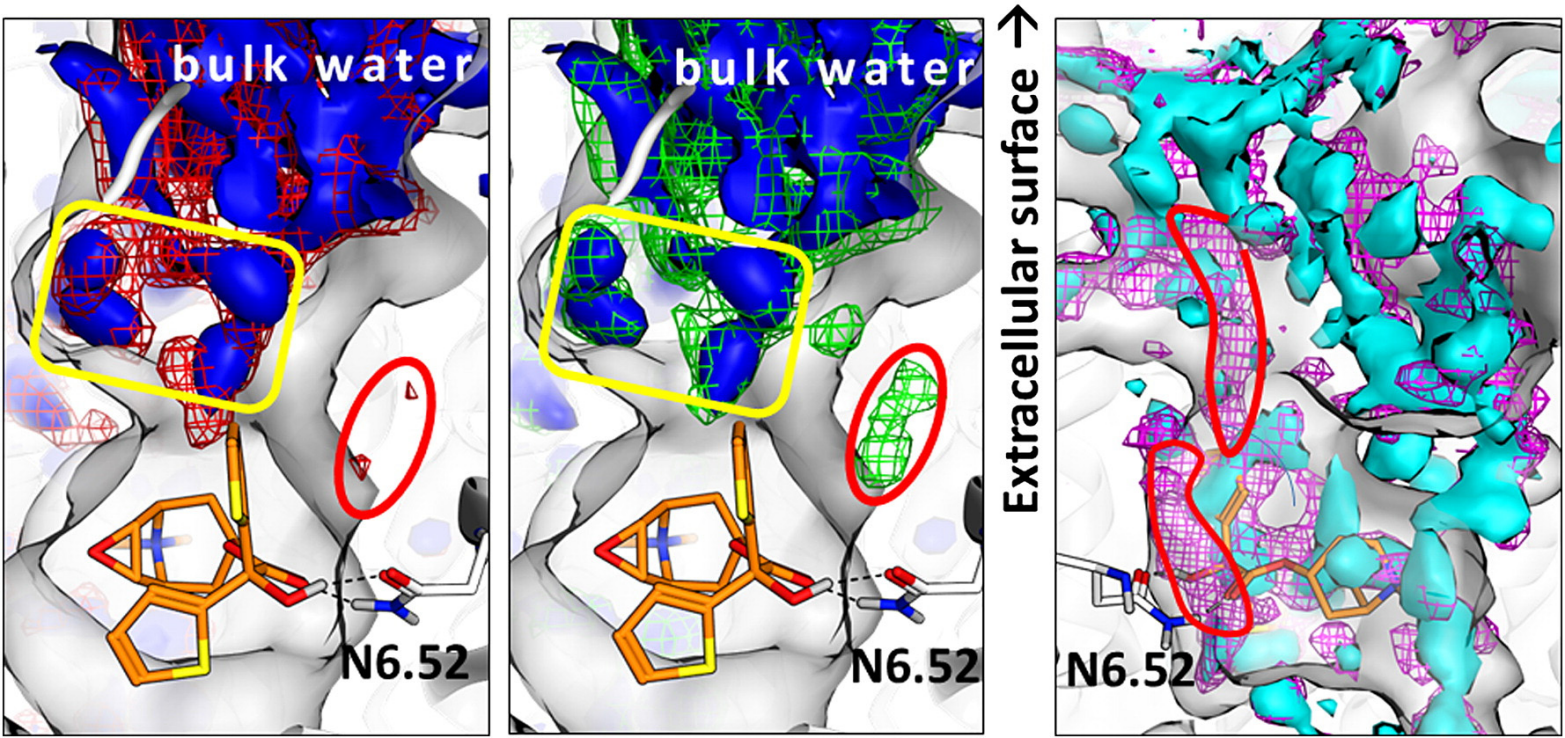
Comparison of the water (oxygen) densities in the tio-WT and the hyd-WT (left panel) and the methyl-WT (middle panel). The tio-WT water densities are displayed as blue solid surfaces, hyd-tio-WT and methyl-tio-WT water densities as red and green mesh, respectively. The most important difference in the water densities is encircled in red. Regions of conserved water networks are marked by a yellow box. Right panel: overlay of apo-WT (magenta mesh) with N508^6.52^A-apo (cyan solid surface) water grids. Regions which are nearly exclusively hydrated in apo-WT are marked by red contours. All grids are overlaid onto the tiotropium bound WT hM3R.

In the previous section we have reported that the channel opening is always accompanied by the approach of water towards N508^6.52^. In this section we are comparing water networks in the simulations by means of calculated water densities. Fig. 10 shows the differences in the tio-WT and hyd-WT/methyl-WT water grids. The most important difference is the occurrence of some water density close to N508^6.52^ in the methyl-WT and also to a smaller extent in the hyd-WT grids. These water locations correspond to those events in which the channel is open and water gets close to the ligand–N508^6.52^ hydrogen bond. Within the ligand binding site no water mediated contacts are observed. Towards the extracellular region, the water densities are becoming more and more bulk-like, however, close to the receptor very distinct water shapes are observed. This means that close to the protein surface water networks are well conserved. In Fig. 8 very strong differences in the channel geometry between apo-WT and apo-N508^6.52^A are shown. The corresponding water grids are also displayed in Fig. 10 (right). In the apo-WT simulation water density is found throughout the channel and also close to N508^6.52^. In contrast the apo-N508^6.52^A simulation shows essentially a “dry” channel and no water density close to A508^6.52^. In other regions of the binding site, i.e., in the tyrosine cage the water grids are similar for both simulations. This means that the lack of N508^6.52^ causes the interruption of the water network connecting the ligand binding site with bulk water. The tiotropium bound simulations (tio-WT and tio-N508^6.52^) do not show any significant difference in the water network densities (Fig. S4, Supplementary material).

To summarize the observations for the water networks: tiotropium binding to either WT or N508^6.52^A-hM3R keeps the channel closed, and no water density is observed in the exit channel. For the modified ligands (methyl-tio and hyd-tio) a distinct water density is observed in the WT-simulation, solvating the ligand-N508^6.52^ hydrogen bond. The N508^6.52^A mutation causes a completely different water density pattern as hardly any water density is found in the exit channel.

### 4.2 Discussion of Case Study 2

Putting together all these findings from the MD simulations we can now understand the differences in the binding dynamics of tiotropium analogs and the effect of the N508^6.52^A mutant. Experimentally we have shown that the removal of one ligand–receptor hydrogen bond leads to overproportionally quick dissociation, no matter if the hydrogen bond partner was removed on the ligand side (hyd-tio, methyl-tio) or on the receptor side (N508^6.52^A). Thus we observed experimentally that the N508^6.52^A mutation of the receptor has exactly the same effect as the removal of the hydroxy group from the ligand. Experimental data however, did not give any hint towards differences in the mechanism which causes accelerated dissociation, whereas insights obtained from MD simulations suggest that there is a major discrepancy between the underlying mechanisms. Upon removal of the hydroxy-group of tiotropium (hyd-tio, methyl-tio) the ligand–receptor hydrogen bond becomes water accessible. This is supposed to have a significant effect on the off-rates. Schmidtke et al. [80] have investigated buried polar atoms in protein binding sites that form ligand–protein hydrogen bonds which are shielded from water. Forming and breaking of this kind of hydrogen bonds lead to an energetically unfavorable transition state because it occurs asynchronously with hydration. In consequence, water-shielded hydrogen bonds are exchanged at slower rates. When tiotropium binds to the receptor the hydrogen bond to N508^6.52^ is water shielded, thus breaking more slowly. For the derivatives hyd-tio and methyl-tio the hydrogen bond is not shielded any longer as water enters into the channel, and the exchange occurs faster. This is now a plausible explanation for the observed rate enhancement upon hydroxy removal from the ligand.

The explanation for the accelerated rate upon N508^6.52^A mutation of hM3R is not that straightforward, because no hydrogen bond needs to be broken and the line of argumentation from above is not applicable. Tiotropium forms an intramolecular hydrogen bond (hydroxy to ester) and no solvation of the hydroxy group of the bound ligand is observed. Indeed in the tio-N508^6.52^A simulation the exit channel is as tightly closed as in the tio-WT simulation. The most striking difference is found in the location of the ligand, where tiotropium frequently progresses into the exit channel in the tio-N508^6.52^A simulation. We thus propose that the first steps of dissociation of tiotropium from N508^6.52^A hM3R do occur through a different mechanism than the dissociation from WT hM3R. In WT a solvation of the hydrogen bond is the first step for dissociation, but in N508^6.52^A hM3R the ligand's motion into the exit channel is not supported by extracellular water. It may be seen as a mechanical event of impinging against the exit channel, which occurs much more often if the hydrogen bond to the receptor is missing.

Obviously the cavity around D148^3.32^ has to be re-solvated upon ligand dissociation (for both cases, WT and N508^6.52^A), but this site is directly connected to the receptor spanning water network which is conserved across class A GPCRs [81]. Therefore there is no need for the synchronous movement of extracellular solvent into the binding site upon ligand dissociation.

To sum up, the MD-simulations provide deeper insight in the mechanisms which cause accelerated dissociation from the M3 receptor. The lack of a hydrogen bond between the ligand and N508^6.52^ of hM3R causes an enhanced off-rate, but the underlying mechanism is different. If the hydroxy group of tiotropium is removed, the hydrogen bond between N508^6.52^ and the ester group of the ligand gets exposed to water and is not shielded any more. However, if N508^6.52^ is mutated to alanine, tiotropium pushes towards the exit channel. In this case solvation of the exit channel does not seem to be important. Without MD simulations we would not be able to elucidate the differences in the dissociation enhancements. The remaining question is if we would have been able to predict behavior like this by MD and thus identify crucial ligand–protein interactions. For systems like the present one there is currently no method available which allows a quantification of the effects. We expect that in the future the application of recently developed simulation and analysis techniques based on Markov State Models together with increased computer power will lead to reliable predictions of off-rates also for highly potent ligands which show slower association rates [36,42,82,83]. For potent ligands, at present, we can use MD simulations only to rationalize the experiments or qualitatively investigate if some modifications of the ligands cause major rearrangements of the receptor or of the water network.

## 5 Summary and outlook

We described two case studies in which MD simulations were employed to investigate the interactions between a GPCR and small molecule ligands. However, both studies were carried out within totally different initial situations: in the case of CCR3, structural information about the ligand–receptor complex had to be generated via homology modeling. We observed that the template which is chosen to derive the homology model had a significant impact on key characteristics of the putative transmembrane binding pocket. For some template/alignment combinations it was even impossible to postulate a reasonable binding mode. With the availability of an X-ray structure of a sufficiently closely related receptor we were able to generate binding hypotheses which were in line with observed species differences and SAR. MD simulations helped to exclude receptor and ligand conformations which might not reflect the most stable state under the conditions that were modeled. At the same time the MD simulations delivered an ensemble of conformations of the receptor–ligand complex which are all accessible. Without additional experimental or computed information it is not possible to predict which configuration is biologically relevant. Experimental information such as structure activity relationship data, species selectivity data as in our case, or – ideally – site-directed mutagenesis data is key to establish a validated binding mode which is suitable for prospective ligand design efforts. Although we were finally able to rationalize species selectivity data and postulate regions for chemical modifications on a very coarse-grained level, it is important to emphasize that the final ligand–receptor model was not accurate enough to assess the impact of chemical modifications on the free energy of binding in a quantitative manner. Also, the accuracy of the model does not allow us to investigate the role of water molecules for ligand binding in detail.

In contrast to the CCR3 case study the computational study of ligand binding to hM3R is based on very detailed experimental (X-ray) structural information of a receptor–ligand complex. This allowed us to investigate the influence of solvation and desolvation and receptor motions on the binding of a congeneric series of small molecules to this receptor on a molecular level. Complemented by site-directed mutagenesis studies, a microscopic model of binding and dissociation could be derived which is in agreement with experimental data collected so far. MD simulations were key to elucidate differences between the behavior of closely related ligands in wild-type and mutated receptors.

In both case studies MD simulations turned out to be instrumental to come up with models which are able to rationalize experimental data in a retrospective fashion. However, the true impact of MD simulations on GPCR drug discovery programs can only be evaluated by prospective applications and subsequent experimental challenging of the predictions. Either synthesis and testing of compounds with proposed structural modifications or measuring ligand binding in mutated receptors will reveal if in silico predictions have been correct. Ideally, a combination of both approaches will be employed. The speed in elucidation of novel GPCR structural information together with further growth in compute power will enable us to carry out computational experiments on a large scale. From a careful analysis of these studies and a rigorous assessment of the agreement or disagreement between computational predictions and experimental outcomes we can hope to derive general rules which can guide the prospective application of MD simulations in GPCR ligand design. This might also be expanded beyond pure binding to e.g. computational modeling of GPCR modulation.

## Figures and Tables

**Table 1:** Systems under investigation in the present study.

	Ligands:	Receptor	Abbreviation of the system
1	Tiotropium (R = OH)	Wild type hM3R	Tio-WT
2	Des-OH tiotropium (R = H) [“hyd-tio”]	Wild type hM3R	Hyd-tio-WT
3	Methyl-des-OH-tiotropium (R = CH_3_) [“methyl-tio”]	Wild type hM3R	Methyl-tio-WT
4	Tiotropium (R = OH)	N508^6.52^A hM3R	Tio-N508^6.52^A
5	None	Wild type hM3R	Apo-WT
6	None	N508^6.52^A hM3R	Apo-N508^6.52^A
