# Supplementary material for: What can we learn from molecular dynamics simulations for GPCR drug design?
Source: Comput Struct Biotechnol J. 2014 Dec 10;13:111–21. doi: 10.1016/j.csbj.2014.12.002 (PMC4334948; doi:10.1016/j.csbj.2014.12.002)
Supplement: Supplementary file 1 — Supplementary material. [file mmc1.docx]

Supporting Information to

**What can we learn from molecular dynamics simulations for GPCR drug design?**

Christofer S. Tautermann, Daniel Seeliger, and Jan M. Kriegl*

Boehringer Ingelheim Pharma GmbH & Co. KG,

Lead Identification and Optimization Support,

Birkendorfer Str. 65, D-88397 Biberach a.d. Riss,

Germany

CCR3 HUMAN 1 ----MTTSLDTVETFGTTSYYDDVGLLCEKADTRALMAQFVPPLYSLVFTVGLLGNVVVVMILIKYRRLRIMTNIYLLNL 76

CCR3 MOUSE 1 MAFNTDEIKTVVESFETTPYEYEWAPPCEKVRIKELGSWLLPPLYSLVFIIGLLGNMMVVLILIKYRKLQIMTNIYLFNL 80

CCR3 RAT 1 MASNEEELKTVVETFETTPYEYEWAPPCEKVSIRELGSWLLPPLYSLVFIVGLLGNMMVVLILIKYRKLQIMTNIYLLNL 80

CCR5 HUMAN 1 --------MDYQVSSPIYDINYYTSEPCQKINVKQIAARLLPPLYSLVFIFGFVGNMLVILILINCKRLKSMTDIYLLNL 72

pdb: 4MBS 1 --------------------------PCQKINVKQIAARLLPPLYSLVFIFGFVGNMLVILILINYKRLKSMTDIYLLNL 54

CCR3 HUMAN 77 AISDLLFLVTLPFWIHYVRGHNWVFGHGMCKLLSGFYHTGLYSEIFFIILLTIDRYLAIVHAVFALRARTVTFGVITSIV 156

CCR3 MOUSE 81 AISDLLFLFTVPFWIHYVLWNEWGFGHYMCKMLSGFYYLALYSEIFFIILLTIDRYLAIVHAVFALRARTVTFATITSII 160

CCR3 RAT 81 AISDLLFLFTVPFWIHYVLWNEWGFGHCMCKMLSGLYYLALYSEIFFIILLTIDRYLAIVHAVLALRARTVTFATITSII 160

CCR5 HUMAN 73 AISDLFFLLTVPFWAHYAA-AQWDFGNTMCQLLTGLYFIGFFSGIFFIILLTIDRYLAVVHAVFALKARTVTFGVVTSVI 151

pdb: 4MBS 55 AISDLFFLLTVPFWAHYAA-AQWDFGNTMCQLLTGLYFIGFFSGIFFIILLTIDRYLAVVHAVFALKARTVTFGVVTSVI 133

CCR3 HUMAN 157 TWGLAVLAALPEFIFYETEELFEETLCSALYPEDTVYSWRHFHTLRMTIFCLVLPLLVMAICYTGIIKTLLRCPS-KKKY 235

CCR3 MOUSE 161 TWGLAGLAALPEFIFHESQDSFGEFSCSPRYPEGEEDSWKRFHALRMNIFGLALPLLVMVICYSGIIKTLLRCPN-KKKH 239

CCR3 RAT 161 TWGFAVLAALPEFIFHESQDNFGDLSCSPRYPEGEEDSWKRFHALRMNIFGLALPLLIMVICYSGIIKTLLRCPN-KKKH 239

CCR5 HUMAN 152 TWVVAVFASLPGIIFTRSQKEGLHYTCSSHFPYSQYQFWKNFQTLKIVILGLVLPLLVMVICYSGILKTLLRCRNEKKRH 231

pdb: 4MBS 134 TWVVAVFASLPNIIFTRSQKEGLHYTCSSHFPYSQYQFWKNFQTLKIVILGLVLPLLVMVICYSGILKTLLRMK-EKKRH 212

CCR3 HUMAN 236 KAIRLIFVIMAVFFIFWTPYNVAILLSSYQSILFGNDCERSKHLDLVMLVTEVIAYSHCCMNPVIYAFVGERFRKYLRHF 315

CCR3 MOUSE 240 KAIRLIFVVMIVFFIFWTPYNLVLLFSAFHRTFLETSCEQSKHLDLAMQVTEVIAYTHCCVNPVIYAFVGERFRKHLRLF 319

CCR3 RAT 240 KAIQLIFVVMIVFFIFWTPYNLVLLLSAFHSTFLETSCQQSIHLDLAMQVTEVITHTHCCINPIIYAFVGERFRKHLRLF 319

CCR5 HUMAN 232 RAVRLIFTIMIVYFLFWAPYNIVLLLNTFQEFFGLNNCSSSNRLDQAMQVTETLGMTHCCINPIIYAFVGEKFRNYLLVF 311

pdb: 4MBS 213 RDVRLIFTIMIVYFLFWAPYNIVLLLNTFQEFFGLNNCSSSNRLDQAMQVTETLGMTHCCINPIIYAFVGEEFRNYLLVF 292

CCR3 HUMAN 316 FHRHLLMHLGRYIPFLPSEKLERTSSV-SPSTAEPELSIVF 355

CCR3 MOUSE 320 FHRNVAVYLGKYIPFLPGEKMERTSSV-SPSTGEQEISVVF 359

CCR3 RAT 320 FHRNVAIYLRKYISFLPGEKLERTSSV-SPSTGEQEISVVF 359

CCR5 HUMAN 312 FQKHIAKRFCKCCSIFQQEAPERASSVYTRSTGEQEISVGL 352

pdb: 4MBS 293 FQ--------------------------------------- 294

Figure S1: Sequence alignment of CCR3 (human and rodent orthologues) with hCCR5 and the GPCR regions of the structural template 4MBS, which is an engineered version of hCCR5. The The seven transmembrane helices in hCCR5 are marked in red.


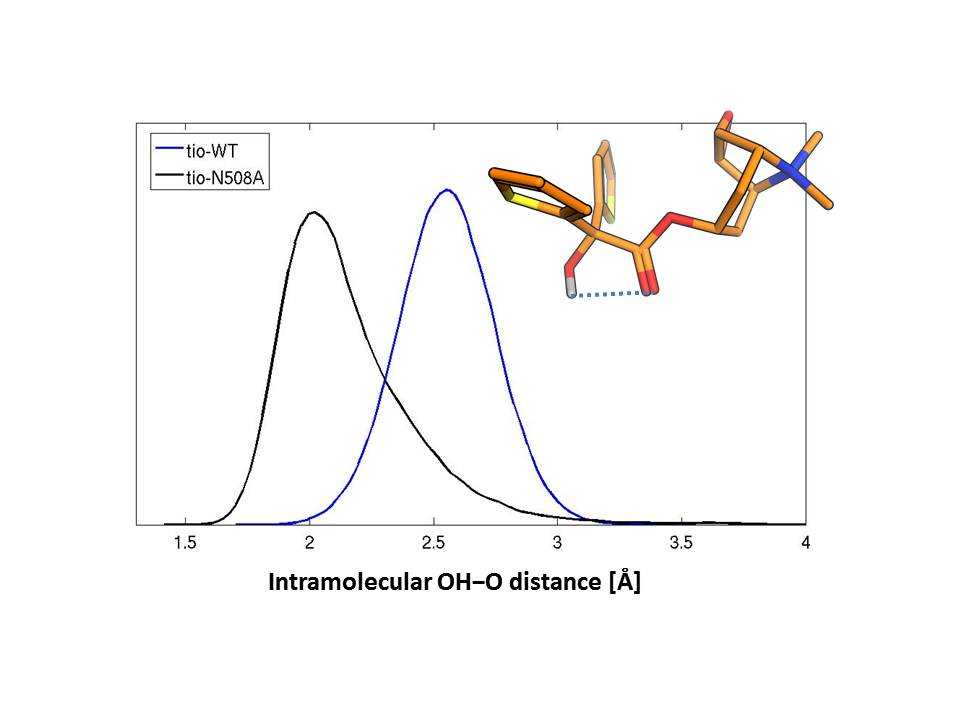


Figure S2: Distribution function of the intramolecular distance of the hydrogen of the OH group to the oxygen of the carbonyl group in tiotropium. Blue: tio-WT simulation, black tio-N508A simulation.


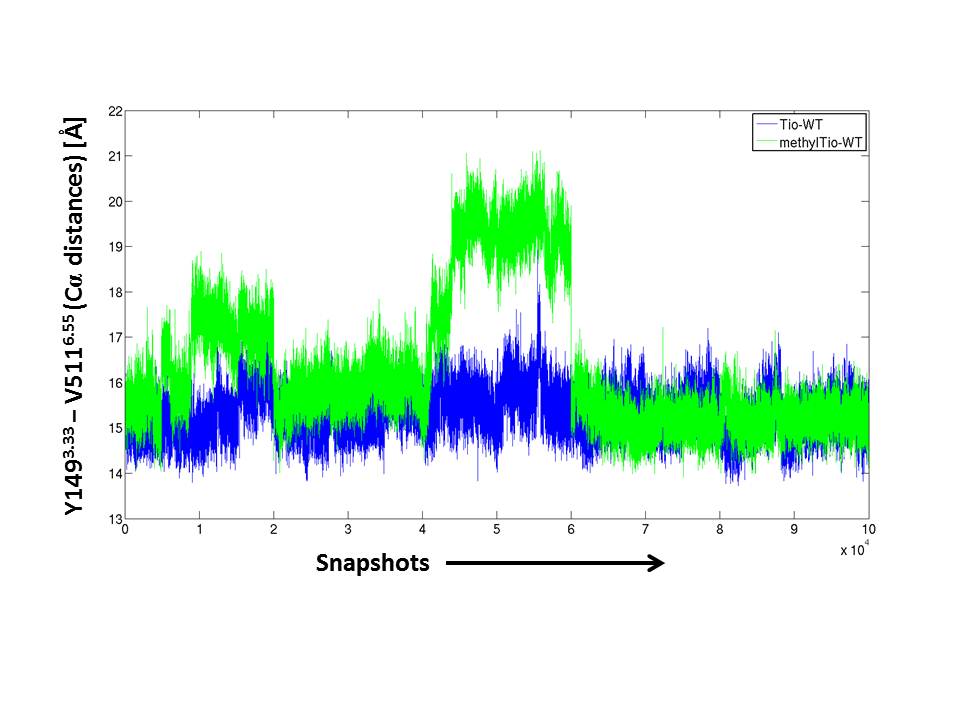


Figure S3: Channel diameters of the M3 entry-channel (distances of the Cα atoms of Y149^3.33^V511^6.55^) during the simulations. Green: methyl-tio-WT and blue: tio-WT.


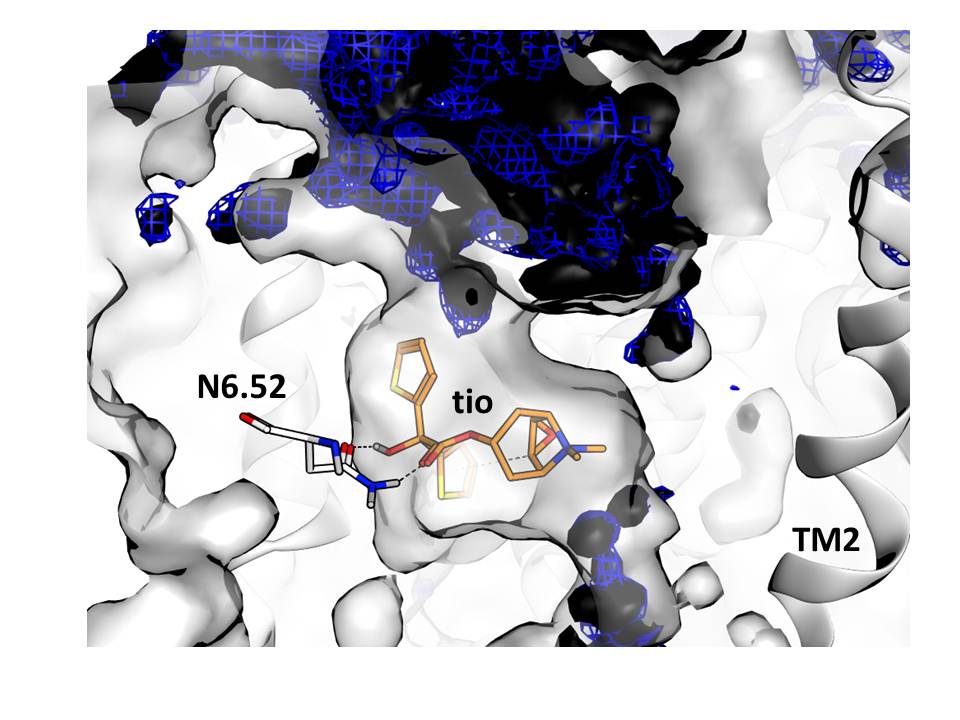


Figure S4: Water densities in the tio-WT (blue mesh) and tio-N508A (black solid surface) simulations.

Figure S5: RMSF values of substructures of the ligands during the MD-simulations. “C_quart and neighbors” corresponds to tiotropiums (or analog) quartenary carbon and the directly bonded heavy atoms. “ester” corresponds to the (C=O)O ester unit and “tropan” corresponds to the N,N-dimethylepoxytropane substructure of the ligands.


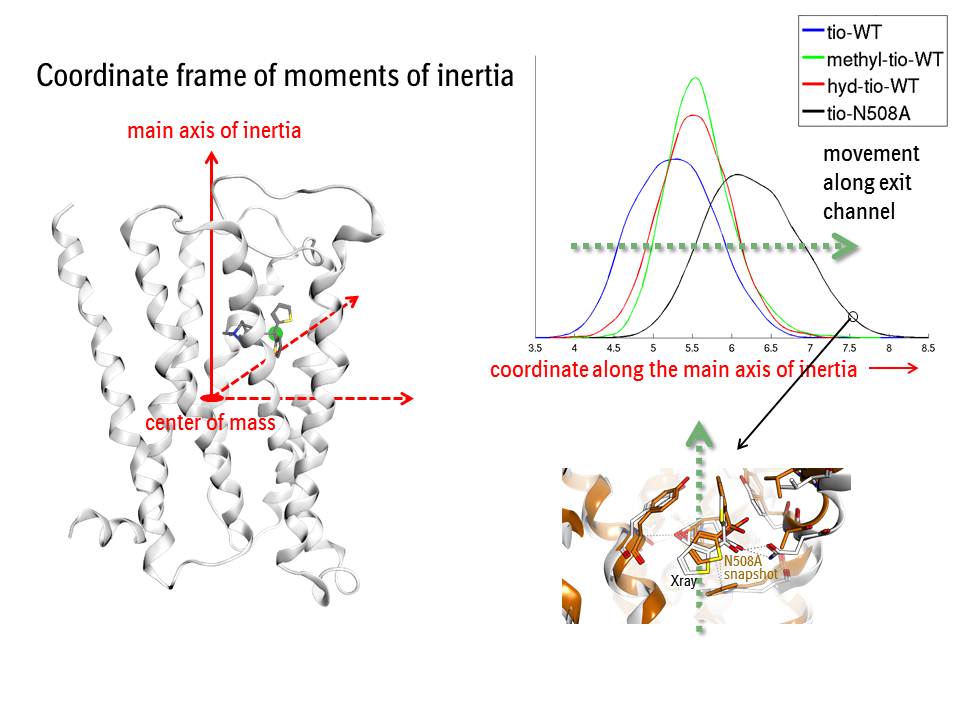


Figure S6: Movement of the quartenary carbon atom in the coordinate system of the main axes of inertia. For tio-N508A the aromatic part of the ligand is significantly displaced towards the extracellular side of the receptor.

|  | Receptor | pKi (membrane binding buffer) | k_off_ [min^-1^] (membrane binding buffer) | pKi  (low ionic strength buffer) | k_off_ [min^-1^] (low ionic strength buffer) |
| --- | --- | --- | --- | --- | --- |
| tiotropium | M3-WT | 10.8 | 4.8E-04 | 10.8 | 3.7E-04 |
| methyl-tio | M3-WT |  |  | 10.3 | 7.9E-03 |
| hyd-tio | M3-WT |  |  | 10.0 | 2.1E-02 |
| tiotropium | M3-N6.52A | 9.1 | 2.8E-01 |  |  |

Table S1: Experimental values for tiotropium (and analoga) binding and dissociation – taken from Tautermann et al. (2013) J Med Chem.14;56(21):8746-56.
